# Comparison of the impact of viral and plant-derived promoters regulating selectable marker gene on maize transformation and transgene expression

**DOI:** 10.1007/s00299-017-2099-y

**Published:** 2017-02-03

**Authors:** Jeffrey Beringer, Wei Chen, Russell Garton, Nagesh Sardesai, Po-Hao Wang, Ning Zhou, Manju Gupta, Huixia Wu

**Affiliations:** 1 Dow AgroSciences, LLC, 9330 Zionsville Road, Indianapolis, IN 46268 USA; 2 Covance, Inc., 8211 SciCor Drive, Indianapolis, IN 46214 USA

**Keywords:** Promoter choice, *Aad-1* and *yfp* genes, Transformation efficiency, Copy number, Gene expression, Maize

## Abstract

**Key message:**

The choice of promoter regulating the selectable marker gene impacts transformation efficiency, copy number and the expression of selectable marker and flanking genes in maize.

**Abstract:**

Viral or plant-derived constitutive promoters are often used to regulate selectable marker genes. We compared two viral promoters, cauliflower mosaic virus (CaMV 35T) and sugarcane bacilliform virus (SCBV) with two plant promoters, rice actin1 (OsAct1) and maize ubiquitin 1 (ZmUbi1) to drive aryloxyalkanoate dioxygenase (*aad*-1) selectable marker gene in maize inbred line B104. ZmUbi1- and OsAct1-containing constructs demonstrated higher transformation frequencies (43.8 and 41.4%, respectively) than the two viral promoter constructs, CaMV 35T (25%) and SCBV (8%). Interestingly, a higher percentage of single copy events were recovered for SCBV (82.1%) and CaMV 35T (59.3%) promoter constructs, compared to the two plant-derived promoters, OsAct1 (40.0%), and ZmUbi1 (27.6%). Analysis of protein expression suggested that the viral promoter CaMV 35T expressed significantly higher AAD-1 protein (174.6 ng/cm^2^) than the OsAct1 promoter (12.6 ng/cm^2^) in T_0_ leaf tissue. When measured in T_2_ callus tissue, the two viral promoters both had higher expression and more variability than the two plant-derived promoters. A potential explanation for why viral promoters produce lower transformation efficiencies but higher percentages of low copy number events is discussed. In addition, viral promoters regulating *aad*-1 were found to influence the expression of upstream flanking genes in both T_0_ leaf and T_2_ callus tissue.

## Introduction

Simple vectors for transformation consist of two transgenes: a selectable marker gene regulated by a constitutive promoter (active continuously in most or all tissues) and a gene-of-interest (GOI) which could be regulated by different types of constitutive, tissue-specific or developmental stage-specific, or inducible (regulated via external chemical or physical applications) promoters (Peremarti et al. 2010). Constitutive promoters controlling the selectable marker gene allow sufficient level of expression, thus facilitating the selective propagation of transformed cells throughout the selection process (Peremarti et al. 2010).

Viruses and plant housekeeping genes are the two major sources of constitutive promoters used to drive selective marker genes (Peremarti et al. 2010). The promoter CaMV 35S from the plant virus, cauliflower mosaic virus, has been identified and widely used (Guilley et al. 1982; Odell et al. 1985; Kay et al. 1987). Modification by adding introns or enhancers containing fragments from either monocots or dicots improve the utility of CaMV promoter in transformation systems of maize and bluegrass (Vain et al. 1996). SCBV promoter from the plant virus, sugarcane bacilliform virus, is active in monocots (Tzafrir et al. 1998), and has been shown to drive high levels of gene expression in banana (Schenk et al. 1999, 2001), sugarcane (Braithwaite et al. 2004) and maize (Davies et al. 2014).

Plant constitutive promoters come from highly conserved families of housekeeping genes required by all cells for basic functions, response to stress or protein synthesis and core metabolism (Peremarti et al. 2010). One such family is for the synthesis of cytoskeletal components, actins and tubulins (McElroy et al. 1990). The rice actin *OsACT1* promoter has strong transient (McElroy et al. 1990, 1991) and stable expression (Zhang et al. 1991). Arabidopsis *ACT2*/*ACT8* (An et al. 1996) and banana *ACT1* (Hermann et al. 2001) also show constitutive or near-constitutive expression. Another family of housekeeping genes is ubiquitins (Christensen et al. 1992; Kawalleck et al. 1993; Christensen and Quail 1996). Among many polyubiquitin promoters from different plant species (Callis et al. 1990; Norris et al. 1993; Garbarino et al. 1995), maize ZmUbi1 has been widely used in monocot transformation, including rice (Cornejo et al. 1993), common and durum wheat (Wu et al. 2003, 2008), barley (Harwood et al. 2000) and maize (Negrotto et al. 2000).

The efficient production of transgenic events is a prerequisite for versatile gene function analysis, proof of concept and trait product development. One of the major factors in the efficiency of transgenic event production is the choice of promoter regulating the selectable marker gene. There are a very limited number of published studies on promoter choices for controlling selectable markers. When the viral promoter CaMV 35S driving selectable marker *nptII* was compared with plant constitutive promoter *Os*ACT1 in maize transformation via particle bombardment, promoter choice was found to affect the transformation efficiency and transgene copy numbers (Prakash et al. 2008). In addition, a viral promoter driving a selectable marker influenced the expression of the neighboring genes, resulting in increased accumulation of transcripts of genes near the site of integration in the genome (Davies et al. 2014), altered expression patterns and phenotypes of transgenic plants (Yoo et al. 2005).

For this study, we chose a diverse set of constitutive promoters with a history of relatively strong and weak expression levels including viral promoters CaMV 35T (modified CaMV 35S) and SCBV, and plant constitutive promoters ZmUbi1 and OsAct1. Four constructs were built with the four different promoters regulating the selectable marker gene *aad-*1 (Wright et al. 2010), and terminating with the maize lipase, ZmLip, 3′ UTR. *Agrobacterium*-mediated transformation was carried out using maize inbred line B104. The effects of different promoters regulating the selectable marker gene *aad-*1were analyzed in transgenic events of these four constructs and it was found that promoter choice has a significant impact on transformation frequency, copy number, gene expression and even the expression of neighboring genes in the expression cassette.

## Materials and methods

### Vector design

Four constructs were built to test the promoters listed in Table 1. Each construct consisted of two gene cassettes: yellow fluorescent protein (*yfp*) visual marker (Shagin et al. 2004) and aryloxyalkanoate dioxygenase (*aad-1*) selectable marker (Wright et al. 2010), arranged in tandem. The *yfp* cassette was located 5′ to the *aad-1* cassette, and separated by a spacer sequence that ranged from 278 to 312 bp depending on the promoter tested. The design of the *yfp* cassette was common to all constructs. It had the *Zea mays* ubiquitin-1 (ZmUbi-1) gene promoter regulating the *yfp* gene, which was terminated by a 3′ untranslated region (UTR) (Ainley et al. 2004) derived from the *Zea mays* Peroxidase-5 gene (ZmPer5 3′ UTR). The *aad-1* cassette consisted of one of the four test promoters in each construct (Table 1) and was terminated by a 3′ UTR derived from the *Zea mays* lipase gene (ZmLip 3′ UTR) (Cowen et al. 2007).

**Table 1:** Promoters driving the selectable marker in the test constructs

Category	Promoter	Origin
Viral promoter	SCBV	Sugarcane bacilliform virus
Viral promoter	CaMV 35T	Cauliflower mosaic virus
Plant promoter (weak)	OsAct1	*Oryza sativa* actin 1 gene
Plant promoter (strong)	ZmUbi1	*Zea mays* ubiquitin-1 gene

The *aad-1* was an aryloxyalkanoate dioxygenase gene from *Sphingobium herbicidovorans* encoding an enzyme with an alpha ketoglutarate-dependant dioxygenase activity which results in metabolic inactivation of the herbicide(s) on which it has enzymatic activity (Wright et al. 2010). The *yfp* was a mutant plant optimized version of natural yellow fluorescent protein from *Phialidium* sp. (Evrogen, Russia, Shagin et al. 2004). It contained a 188 bp LS1 intron (Vancanneyt et al. 1990) derived from the potato gene encoding light inducible leaf-/stem-specific protein.

The CaMV 35T promoter of 993 bp was a modified version of the CaMV 35S promoter and consisted of the CaMV 35S promoter and enhancer of 599 bp. Fused to it at the 3′ end was a maize streak virus (MSV) coat protein gene 5′ UTR sequence interrupted by intron-6 of the maize alcohol dehydrogenase-1 (*adh-1*) gene (Merlo and Folkerts 2000). The sugarcane bacilliform virus (SCBV) promoter of 1429 bp was enhanced by fusing the SCBV promoter as described previously (Olszewski et al. 2002) with a 5′ UTR sequence derived from a MSV coat protein gene, which was interrupted by intron-6 of the *adh-1* gene. The maize Ubi1 promoter of 1991 bp consisted of the 5′ UTR and associated intron (1014 bp) derived from the *Zea mays* ubiquitin-1 (ZmUbi-1) gene (Christensen et al. 1992). The OsAct1 promoter of 1397 bp comprised the 5′ UTR and associated intron (553 bp) derived from the *Oryza sativa* actin 1 (OsAct1) gene (McElroy and Wu 1997).

Each promoter was cloned upstream of the *aad-*1 coding sequence in otherwise identical binary vectors which also carried a *yfp* visual marker gene cassette as described above. The distance between the 3′ end of the four promoters, OsAct1, SCBV, CaMV 35T and ZmUbi1, and the ATG start codon of *aad-1* varied between 2, 10, 42 and 40 nucleotides, respectively. A modified EHA105 *Agrobacterium* strain harboring the binary vectors was used to transform immature embryos. The *Agrobacterium* strain was a *RecA*-deficient EHA105 strain and harbored a helper plasmid containing a copy of the virulence gene operons VirB, VirC,VirD and VirG (Merlo et al. 2015).

### *Agrobacterium*-mediated transformation of maize

Modifications were made based on the methodology using maize inbred B104 (Frame et al. 2002, 2006). All subsequent media used in the following transformation procedure are described in Miller (2013), if not specified.

*Agrobacterium* cultures were streaked from glycerol stocks onto solidified AB minimal medium (Chilton et al. 1974) for 3 days and then on YEP medium for 1 day before being used for inoculation. On the day of an experiment, 1–2 loops of *Agrobacterium* from the YEP plate were suspended in 15 ml of the infection medium/acetosyringone mixture (200 µM acetosyringone) inside a sterile, disposable, 50-ml centrifuge tube and the optical density of the solution at 600 nm (O.D._600_) was measured in a spectrophotometer. The suspension was then diluted down to 0.25–0.35 O.D._600_ using additional infection medium/acetosyringone mixture. The tube of *Agrobacterium* suspension was then placed horizontally on a platform shaker set at about 75 rpm at room temperature for between 1 and 4 h before use.

Ears from *Zea mays* cultivar B104 were harvested 10–12 days post pollination. Harvested ears were de-husked and surface-sterilized by immersion in a 20% solution of commercial bleach (Ultra Clorox® Germicidal Bleach, 6.15% sodium hypochlorite) and two drops of Tween 20, for 20 min, followed by three rinses in sterile, deionized water inside a laminar flow hood. Immature zygotic embryos were aseptically excised from each ear and distributed into one or more micro-centrifuge tubes containing 2.0 ml of *Agrobacterium* suspension.

Upon completion of the embryo isolation the tube of embryos was closed and placed on a rocker platform for 5 min. The contents of the tube were then poured out onto a plate of co-cultivation medium and the liquid *Agrobacterium* suspension was removed with a sterile, disposable pipette. Using a microscope, embryos were oriented with the scutellum facing up. The plate was then closed, sealed with 3M Micropore Medical Tape (3M, Saint Paul, USA), and placed in an incubator at 25 °C for 3 days.

Following the co-cultivation period, embryos were transferred to resting medium, and incubated for 7–10 days. Callused embryos were then transferred onto selection medium I with 100 nM haloxyfop. Seven days later, they were transferred to selection medium II with 500 nM haloxyfop. Two weeks later, resistant calli were moved to pre-regeneration medium. Regenerating calli were then transferred to regeneration medium I in Phytatrays™ (Sigma–Aldrich®, Saint Louis, USA) and incubated at 28 °C with 16 h light/8 h dark per day at approximately 150 μmol m^−2^ s^−1^ photosynthetically active radiation (PAR) for 7–14 days or until shoots developed (Davies et al. 2014). Small shoots with primary roots were then isolated and transferred to regeneration medium II. For each construct, between 25 and 35 T_0_ plants originating from different embryos were sampled and analyzed for *aad-*1 and *yfp* gene copy numbers by hydrolysis probe assays. Events found to be positive for both genes and low (1–2) in copy number were advanced to the greenhouse.

Plants were transplanted from phytatrays to small pots (T. O. Plastics, 3.5″ SVD, 700,022 C), filled with growing medium (Premier Tech Horticulture, ProMix BX, 0581 P), and covered with humidity domes. Plants were placed in a growth chamber (28 °C day/24 °C night, 16 h light/8 h dark per day, 50–70% relative humidity, 200 µmol m^−2^ s^−1^ PAR) until reaching V3–V4 stage. This aided in acclimating the plants to soil and a wider range of temperatures. Plants were then moved to the greenhouse (high light limit: 1200 µmol m^−2^ s^−1^ PAR, 16 h day length, 27 °C day/24 °C night) and transplanted from the small pots to 5.5 inch pots to grow to maturity.

T_0_ plants were reciprocally crossed with B104 to obtain T_1_ seeds for generating T_2_ embryos for callus induction. Resulting T_1_ seeds were either null or hemizygous for the genes of interest. These seeds were planted and leaf punches were taken from emerging shoots to analyze for *aad*-1 and *yfp* gene copy numbers. Plants that were hemizygous for both *aad*-1 and *yfp* genes were advanced to the next generation through reciprocal crossing with B104. Immature embryos from these pollinations were harvested when embryo sizes were in the 1.8–2.2 mm range and plated on resting media for 7 days. Embryos were selected via YFP expression under a fluorescence microscope prior to transfer to resting medium with no selection agent for a further 2–3 weeks to generate a sufficient amount of callus to sample for protein expression.

### Genomic DNA isolation

At T_0_, leaf tissue was collected from rooted putative transgenic plants before transplanting to soil to screen for low copy, simple events. At T_1_, 1 cm leaf punches were collected at the V4 stage for zygosity analysis. DNA was extracted with a Qiagen MagAttract 96 DNA Plant Core Kit (Qiagen, Gaithersburg, MD) using KingFisher magnetic particle processors (Thermo Scientific, Waltham, MA) following manufacture’s recommendation.

### Quantitative PCR

Two genes-of-interest (GOI), *aad*-1 and *yfp*, were analyzed with hydrolysis probe assay. GOI detection was performed on a LightCycler^®^480 system (Roche Applied Science, Indianapolis, IN). Biplex quantitative PCR assay with GOI and a low copy maize endogenous gene, *invertase* (GenBank™ Accession No. U16123), was set up accordingly (Kumar et al. 2015). The assay was prepared with 1X LightCycler^®^ 480 Probes Master in a 10 µL of volume reaction containing 0.1% of polyvinylpyrrolidone (PVP 40), 0.4 µM of each primer and 0.2 µM of each probe (For *aad*-1 detection: 5′ oligo: TGTTCGGTTCCCTCTACCAA; 3′ oligo: CAACATCCATCACCTTGACTGA; Probe: 6FAM-CACAGAACCGTCGCTTCAGCAACA-MGB; For *yfp* detection: 5′ oligo: CGTGTTGGGAAAGAACTTGGA; 3′ Oligo: CCGTGGTTGGCTTGGTCT; Probe: 6FAM-CACTCCCCACTGCCT-MGB; For *Invertase*: 5′ oligo: TGGCGGACGACGACTTGT; 3′ oligo: AAAGTTTGGAGGCTGCCGT; Probe: Hex-CGAGCAGACCGCCGTGTACTT-BHQ). After a 10 min hot start at 95 °C, amplification was conducted with 10 s at 95 °C for denaturation, 35 s at 58 °C for annealing and 1 s at 72 °C with fluorescence acquisition. Data were analyzed with ΔΔCt methodology (LightCycler^®^ 480 Software Release 1.5.0).

### Protein extraction

Approximately 50–100 mg of callus tissue or leaf tissue was extracted with 600 μL of callus extraction buffer (PBS containing 0.05% Tween 20, 5 mM EDTA, 5 mM DTT) and 1% of protein inhibitor cocktail (Research Products International Corp, P51200) or leaf extraction buffer (PBS containing 0.05% Tween 20 and 0.5% BSA), correspondingly, using the Geno/Grinder (Model 2010; Spex Sampleprep) to disrupt the leaf tissue. Extracts were centrifuged, and the supernatant containing the soluble proteins was used for ELISA. Pierce^™^ 660 nm protein assay was performed on leaf or callus extracts to determine total soluble protein (tsp) of each sample for normalizing data.

### AAD-1 ELISA

An AAD-1–specific monoclonal antibody (2 μg/mL) was immobilized on microtiter wells to capture AAD-1 protein in plant extracts (Wright et al. 2010). Various concentrations of plant extracts were incubated in the wells. To prepare a standard curve, different concentrations of purified AAD-1 proteins (32, 24, 16, 8.0, 4.0, 2.0, 0.1, and 0 ng/mL) were also put into the microtiter wells on the same assay plate in duplicates. Biotinylated anti–AAD-1 monoclonal antibody (1 μg/mL) solution in PBST was added to each well and incubated for 1 h to detect the bound AAD-1 protein. After washing three times with PBS with 0.05% Tween 20 (PBST), neutravidin–alkaline phosphatase (AP) conjugate (Pierce Biotech) was added to each well and incubated for 30 min. After another wash step, pNPP (*p*-nitrophenyl phosphate) solution (AP substrate; 100 μL) was added, and after the 45-min incubation, the absorbance at 405 nm was measured using a microtiter plate reader. The concentration of proteins in plant extracts was extrapolated from a standard curve developed on the same plate.

### YFP ELISA

Mouse anti-YFP monoclonal antibody (Origene # TA150028) (1 μg/mL) was immobilized on microtiter wells to capture YFP protein in plant extracts (described above). Various concentrations of plant extracts were incubated in the wells. To prepare a standard curve, different concentrations of YFP protein (Axxora EVN-FP651-C100) at 2.00, 1.00, 0.50, 0.25, 0.125, 0.0625, 0.03125, and 0 ng/mL were also put into the microtiter wells on the same assay plate in duplicates. The plate was incubated for 1 h and washed three times with PBS with 0.05% Tween 20 (PBST). Rabbit anti-YFP polyclonal (Axxora #EVN-AB603-C100) (1 μg/mL) solution in PBST containing 0.5% BSA was added and incubated for 1 h. After another wash step, the plate was incubated with a horseradish peroxidase (HRP) conjugated secondary antibody goat anti-rabbit IgG antibody (Pierce #31463) (20 ng/mL) for 30 min. To detect the bound YFP protein, 100 μL of the substrate TMB (3,3′,5,5′-tetramethylbenzidine) (Pierce # 34028) was added to each well and incubated for 10 min after washing three times The color reaction was stopped by 100 µL of 0.4 N H_2_SO_4_ and the absorbance at 450 nm was measured using a microtiter plate reader. The concentration of proteins in plant extracts was extrapolated from a standard curve developed on the same plate.

### Data analysis

Statistical tests were carried out in the JMP Pro 11.2.0 Statistical Discovery software package (SAS Institute Inc.). Differences in mean protein expression were analyzed first by applying the Brown–Forsythe test to determine if the variances were equal. If there was no significant difference between group variances, ANOVA was used to see if the group means were different. If ANOVA was significant, the all pairs Tukey–Kramer test was used to determine which means were different. In the cases where the Brown–Forsythe test determined the variances were unequal, a Welch ANOVA was used to determine if the group means were different.

## Results

### The effect of promoter regulating selectable marker on transformation frequency is varied

**Fig. 1:**
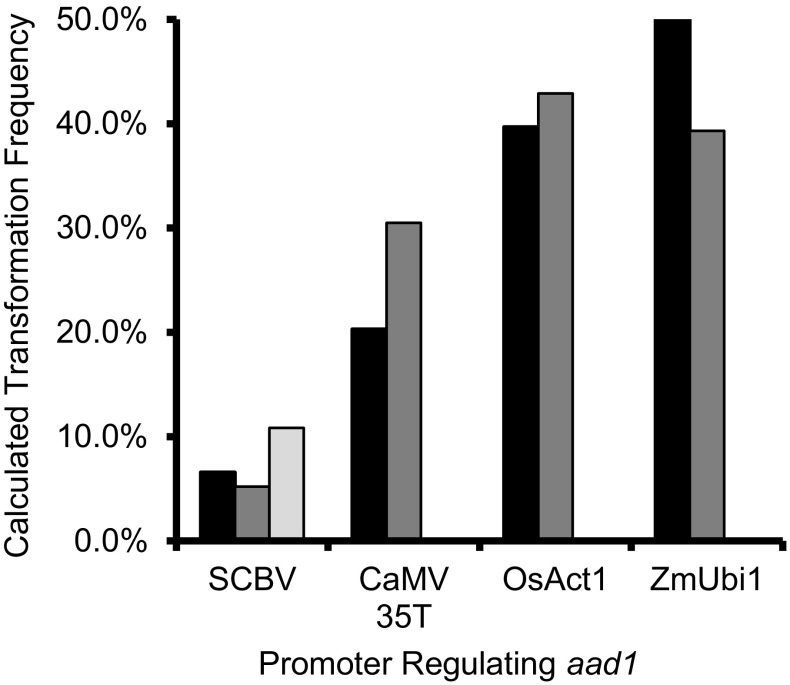
The effect of different promoters regulating the selectable marker gene *aad-*1 on maize B104 transformation frequency. Calculated transformation frequency is the percentage of treated embryos which produced at least one viable shoot after selection on media containing the herbicide haloxyfop. To reduce variation, the transformation experiments for each repeat were carried out on the same day and all transfers used the same media lots

Transformation frequency was calculated by dividing the number of embryos that produced at least one viable T_0_ shoot by the total number of embryos treated. Typically, type I callus produced multiple shoots, but only one shoot per embryo was isolated. This was done to ensure that all plants isolated were from independent events, but this practice will lead to a slight underestimation of transformation frequency because embryos are capable of producing shoots from more than one event. For each construct, 534–665 embryos were used, 2–3 repeated transformation experiments were carried out, and 52–261 events generated, giving average transformation efficiency ranging from 8 to 44% (Fig. 1). The constitutive promoters ZmUbi1 and OsAct1 demonstrated higher transformation frequencies (43.8 and 41.4%, respectively) as compared with the viral promoters CaMV 35T and SCBV (25 and 8%, respectively).

### Percentage of events with a single copy of the transformation cassette varies by promoter

**Fig. 2:**
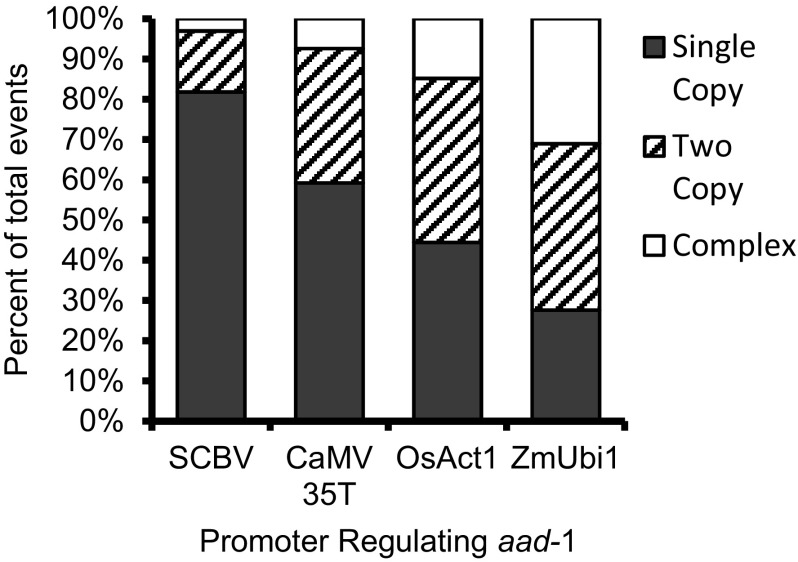
The effect of different promoters regulating the selectable marker gene *aad*-1 on copy number of both *aad*-1 and *yfp* genes. Leaf punches were taken from T_0_ shoots immediately prior to transplanting to soil. Events were categorized based on qPCR as containing a single copy, two copies, or three or more copies of the trangenes which is labeled complex

A random selection of total events produced, around 30 events per construct, were analyzed for copy number based on qPCR. Copy number for the GOI was estimated by comparison of LightCycler^®^480 outputs of target/reference gene values for unknown samples to target/reference gene values of known copy number standards. Events were categorized as single copy, two copies, or complex (containing three or more copies of the transformation cassette). For rare events containing different copy numbers for *aad*-1 and *yfp* genes, including a few with one copy of *aad-1* but no *YFP*, all were eliminated. Figure 2 shows the copy number distribution of the analyzed events positive for both transgenes. Frequency of single copy events by promoter was inversely correlated with transformation frequency. Both viral promoters, SCBV and CaMV 35T, yielded higher percentages of single copy events, 82.1 and 59.3%, respectively, than two plant promoters, OsAct1 (40.0%), and ZmUbi1 (27.6%). In the category of events with complex copy numbers, SCBV had the lowest (3.6%) numbers, followed by CaMV 35T (7.4%), OsAct1 (16%), and ZmUbi1 (31%). In summary, the events from the constructs carrying viral promoters were predominantly single copy events.

### The choice of selectable marker promoter may affect flanking gene expression

To further investigate the different transformation outcomes from viral and plant promoters, transgene protein expression was analyzed. At the V5 growth stage, sets of one and two copies of T_0_ plants were assayed for both AAD-1 and YFP protein levels. Mean protein levels are shown in Tables 2 and 3. The mean AAD-1 expression from the construct using CaMV 35T as promoter driving *aad*-1 (174.6 ng/cm^2^) was the highest, followed by ZmUbi1 (120.0 ng/cm^2^), SCBV (108.3 ng/cm^2^), and OsAct1 was the lowest (12.6 ng/cm^2^). The Brown–Forsythe test indicates the group variances are equal. ANOVA shows that the group means are different and the Tukey–Kramer test shows which means are different (Table 2). These statistical data suggested that the viral promoter CaMV 35T drove significantly higher AAD-1 expression (174.6 ng/cm^2^) than the OsAct1 promoter (12.6 ng/cm^2^).

**Table 2:** Comparison of AAD-1 protein expression in V5 leaf of T_0_ events generated from four different constructs using different promoters regulating *aad*-1 selectable marker gene

Promoter driving *aad*-1	Number of plants tested	Mean AAD-1 protein level (ng/cm^2^)	Std dev	Brown–Forsythe test (Prob > F)	ANOVA (Prob > F)	All pairs, Tukey–Kramer (α = 0.05), connecting letters report^a^
CaMV 35T	21	174.6	152.6	0.0703	0.0385	A
ZmUbi1	15	120.0	57.7			A B
SCBV	25	108.3	247.9			A B
OsAct1	16	12.6	9.4			B

^a^Promoters not connected by the same letter have significantly different mean AAD-1 protein levels

**Table 3:** Comparison of YFP protein expression in V5 leaf of T_0_ events generated from four different constructs using different promoters for *aad*-1 selectable marker gene

Promoter driving *yfp*	Promoter driving *aad-1*	Number of plants tested	Mean YFP protein level (ng/cm^2^)	Std dev	Brown–Forsythe test (Prob > F)	Welch ANOVA (Prob > F)
ZmUbi1	CaMV 35T	21	77.3	47.1	0.0003	<0.0001
ZmUbi1	ZmUbi1	15	44.0	26.6		
ZmUbi1	SCBV	25	24.7	15.6		
ZmUbi1	OsAct1	16	42.5	24.4		

To investigate if the selectable marker promoter influences the upstream flanking genes, we further analyzed the *yfp* gene expression. The *yfp* gene in all four constructs was driven by the same promoter and was expected to display a comparable level of expression among the events generated from all four different constructs. Surprisingly, Table 3 showed varied level of expression for different constructs; CaMV 35T gave the highest (77.3 ng/cm^2^), followed by ZmUbi1 (44.0 ng/cm^2^), OsACT1 (42.5 ng/cm^2^), and SCBV (24.7 ng/cm^2^). Given the unequal group variances, Welch ANOVA was used and determined that there are significant differences in mean YFP protein expression among the four constructs.

### The effect of selectable marker promoter on gene expression of *aad-1* and neighboring gene (*yfp*) at the callus level

**Fig. 3:**
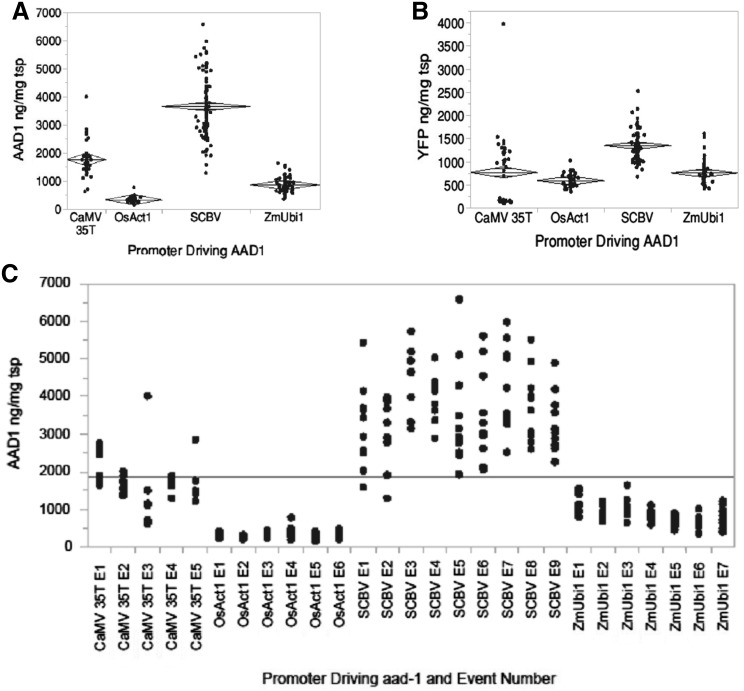
Effect of different promoters regulating the selectable marker gene on protein expression in callus derived from T_2_ embryos. All callus events were from embryos prescreened for YFP expression and are hemizygous for both transgenes. Protein was extracted from 50 to 100 mg of callus tissue using a PBS-based buffer, total soluble protein (tsp) was determined with the Pierce 660 nm protein assay, and ELISA was used to quantify AAD1 and YFP proteins using monoclonal antibodies. **a, b** AAD-1 and YFP (repectively) protein expression in T_2_ callus derived from constructs containing CaMV35T, OsAct1, SCBV and ZmUbi1 driving the *aad*-1 selectable marker gene. The *centerline of the diamonds* indicates the group mean. Expression data were from between 5 and 9 events per promoter and each data point represents a unique callus derived from a single embryo. Each event was represented by at least five and as many as 12 different calli. **c** AAD1 protein expression plotted by event and showing the callus to callus variability within each event

To investigate the effect of promoter on the transformation process, the expression level of selectable marker at callus level was also determined. AAD-1 and YFP protein expression were measured in individual callus grown from immature embryos of the T_2_ generation. Each data point in Fig. 3 represented one sample, of approximately 0.5 mL packed cell volume, from a callus derived from a different embryo. Protein concentrations are expressed in ng/mg total soluble protein. Group means were differentiated using the Tukey–Kramer test. The two viral promoters (SCBV and CaMV 35T) both had higher expression and more variability than the plant promoters (Fig. 3). Mean AAD-1 protein expression levels across all events tested for each construct were found to be significantly different (Brown–Forsythe test for equal variance: *F* = 48.7623, *P* = 0.0000; Welch ANOVA: *F* = 377.3448, *P* < 0.0001). YFP protein expression at callus level from the same construct being more uniform than AAD-1 expression might be expected due to the same promoter driving the *yfp* gene in each construct. However, mean YFP expression was significantly different among the four constructs (Brown–Forsythe test: *F* = 28.5766, *P* = 0.0000; Welch ANOVA: *F* = 120.5854, *P* < 0.0001) (Fig. 3b).

## Discussion

Transformation frequencies appeared to skew lower for the two promoters of viral origin and higher for the promoters of plant origin. It should be noted that transformation frequency is not just a measurement of transgene integration efficiency but results from a combination of integration and stable expression of transgenic protein over the course of the entire 6 week selection period. Therefore, it was necessary to analyze events for gene copy number and to choose simple copy events to compare protein expression at both the in vitro callus stage and in T_0_ leaf tips.

The percentage of single copy events carrying the viral promoters was higher than the percentage for events carrying plant promoters. However, overall transformation frequency was higher using the plant promoters. The trends shown in Figs. 1 and 2 suggest an inverse association between transformation frequency and single copy event ratio: low transformation frequency and high single copy production rate using viral promoter. Increased copy number of transgenes has been shown to have negative effects, leading to co-suppression of the transgene (Napoli et al. 1990; Van der Krol et al. 1990). The low transformation frequency for constructs in which the viral promoter was used may have resulted from the low survival rate of multicopy events due to suppression of the selectable marker expression. At the same time, this could have led to the high ratio of single copy events produced. The silencing of AAD-1 expression in multicopy events carrying the viral promoters may account for the reduced numbers of the events surviving from the selection procedures. The single copy events carrying viral promoters demonstrated much higher AAD-1 expression, as compared with the plant promoters. Thus, multicopy events carrying the viral promoter may trigger the silencing machinery via epigenetic mechanisms to knock down/out the expression level of the selectable marker, leading to lethality in the selection process. The RNA-mediated transgene silencing using the viral 35S promoter has been previously reported (Pinto et al. 1999). Furthermore, multicopy-induced transgene silencing may be associated with transcriptional gene silencing via DNA methylation (Kumpatla and Hall 1998).

The optimal promoter for a commercial event production system requires the best combination of transformation frequency and desired event quality (i.e. single copy event). The expected number of single copy events can be calculated by multiplying the numbers of treated embryos by the calculated transformation frequency and the percentage of single copy events obtained from each promoter. The construct containing CaMV 35T may produce the most single copy events followed by the construct containing OsAct1 (3). Both promoters are in the mid-range of transformation frequency and percent single copy events. Selection of the optimal promoter to enhance commercial event production may therefore require the balance of transformation frequency and the single-copy production rate.

Because *yfp* was driven by ZmUbi1 in all constructs and *yfp* and *aad-1* are arranged in tandem, we expected YFP expression to be fairly uniform across constructs. However, analysis indicated that the construct containing CaMV 35T regulating *aad*-1 produced significantly higher YFP expression than seen in the other constructs. YFP expression was also measured in the T_2_ calli. As with the plant expression data we expected a fairly uniform expression level across constructs but we observed the highest expressing promoter regulating *aad*-1 (SCBV) also boosted the expression of the neighboring upstream *yfp* gene. Previous studies also observed the potential effects on the flanking gene expression influenced by the flanking viral promoter driving the selectable marker: the gene was activated by the flanking CaMV 35S enhancer and such effects may reach up to 4.3 kb upstream or downstream from the insertion site (Tani et al. 2004). In addition, the read-through mRNA transcripts may have potential to influence the transgene expression (Herr et al. 2006). However, since all the *yfp* was driven by the same promoter and 3′UTR terminator, the varied expression level may be resulted from other cause but not aberrant mRNA. Altogether, it suggests the potential challenges to obtain the predictable expression with constructs containing viral promoters while the two flanking transgenes may mutually influence each other. The CaMV 35T promoter may play a role of enhancer to boost the expression of the neighboring upstream gene. This further implied an important consideration when designing constructs with stacked traits where the expression of each trait must be within an optimal range.

A further consideration when choosing a promoter to express a selectable marker gene is its performance in callus during the in vitro culture stage. Expression of the selectable marker gene must be maintained throughout callus proliferation, embryogenesis, regeneration, and shoot elongation culture phases. Figure 3c illustrates the AAD-1 expression variability between different calli derived from the same transgenic event. The viral promoters produced a wider range of expression which makes them less predictable and more challenging to include in a stacked trait construct. If a specific range of expression was required for a particular gene, many more events would be required to find one with the optimal expression level. Despite relatively low levels of AAD-1 driven by OsAct1 in callus tissue, transformation frequency for the OsAct1 construct was quite high, indicating only a small amount of protein is required to confer resistance to haloxyfop in culture.

## Conclusion

Transformation frequency and the percentage of single copy events varied with the type of promoter regulating the selectable marker in constructs. Two plant promoters, ZmUbi1 and OsAct1 resulted in higher transformation frequencies than the two viral promoters, CaMV 35T and SCBV. In contrast, two viral promoters gave higher percentage of single copy events than the two plant promoters. Analysis of protein expression revealed that promoters which drove high expression of the selectable marker gene did not result in higher transformation frequencies and low protein expression was not associated with a reduction in transformation frequency. These results suggest that the choice of promoter has a significant effect on maize transformation efficiency, transgene integration copy number, and most importantly, gene expression of not only the selectable marker gene, but also the upstream gene-of-interest in the transformation vector. When designing constructs for functional gene expression, the fact that a viral promoter could affect flanking gene expression needs to be taken into consideration.

### **Author contribution statement**

JB led maize transformation, data analysis for transformation, copy number and gene expression and contributed to experiment design and manuscript writing. WC provided DNA analysis methodology, led DNA analysis and contributed to writing. RG led protein analysis. NS contributed to experiment design, constructed vectors. PW contributed to discussion of gene silencing and writing. NZ provided protein analysis methodology and writing. MG contributed to experiment design, monitored the experiment progress and provided regulatory element descriptions. HW conceived, contributed to experiment design and led the manuscript writing. All authors read and approved the manuscript.
